# Identification and Design of Novel Potential Antimicrobial Peptides Targeting Mycobacterial Protein Kinase PknB

**DOI:** 10.1007/s10930-024-10218-9

**Published:** 2024-07-16

**Authors:** Hemchandra Deka, Atul Pawar, Monishka Battula, Ayman A. Ghfar, Mohamed E. Assal, Rupesh V. Chikhale

**Affiliations:** 1 SilicoScientia Private Limited, Nagananda Commercial Complex, No. 07/3, 15/1, 18th Main Road, Jayanagar 9th Block, Bengaluru, 5600413 India; 2 Department of Bioinformatics, Rajiv Gandhi Institute of IT and Biotechnology, Bharati Vidyapeeth Deemed to be University, Pune-Satara Road, Pune, India; 3 Chemistry Department, College of Science, King Saud University, Riyadh, 11451 Saudi Arabia; 4 Department of Pharmaceutical and Biological Chemistry, School of Pharmacy, University College London, Brunswick Square, London, UK

**Keywords:** Serine/threonine Protein Kinase (STPK), ADCP, Gromacs, Elastic Network Model (ENM)

## Abstract

Antimicrobial peptides have gradually gained advantages over small molecule inhibitors for their multifunctional effects, synthesising accessibility and target specificity. The current study aims to determine an antimicrobial peptide to inhibit PknB, a serine/threonine protein kinase (STPK), by binding efficiently at the helically oriented hinge region. A library of 5626 antimicrobial peptides from publicly available repositories has been prepared and categorised based on the length. Molecular docking using ADCP helped to find the multiple conformations of the subjected peptides. For each peptide served as input the tool outputs 100 poses of the subjected peptide. To maintain an efficient binding for relatively a longer duration, only those peptides were chosen which were seen to bind constantly to the active site of the receptor protein over all the poses observed. Each peptide had different number of constituent amino acid residues; the peptides were classified based on the length into five groups. In each group the peptide length incremented upto four residues from the initial length form. Five peptides were selected for Molecular Dynamic simulation in Gromacs based on higher binding affinity. Post-dynamic analysis and the frame comparison inferred that neither the shorter nor the longer peptide but an intermediate length of 15 mer peptide bound well to the receptor. Residual substitution to the selected peptides was performed to enhance the targeted interaction. The new complexes considered were further analysed using the Elastic Network Model (ENM) for the functional site’s intrinsic dynamic movement to estimate the new peptide’s role. The study sheds light on prospects that besides the length of peptides, the combination of constituent residues equally plays a pivotal role in peptide-based inhibitor generation. The study envisages the challenges of fine-tuned peptide recovery and the scope of Machine Learning (ML) and Deep Learning (DL) algorithm development. As the study was primarily meant for generation of therapeutics for Tuberculosis (TB), the peptide proposed by this study demands meticulous invitro analysis prior to clinical applications.

**Graphical Abstract:**

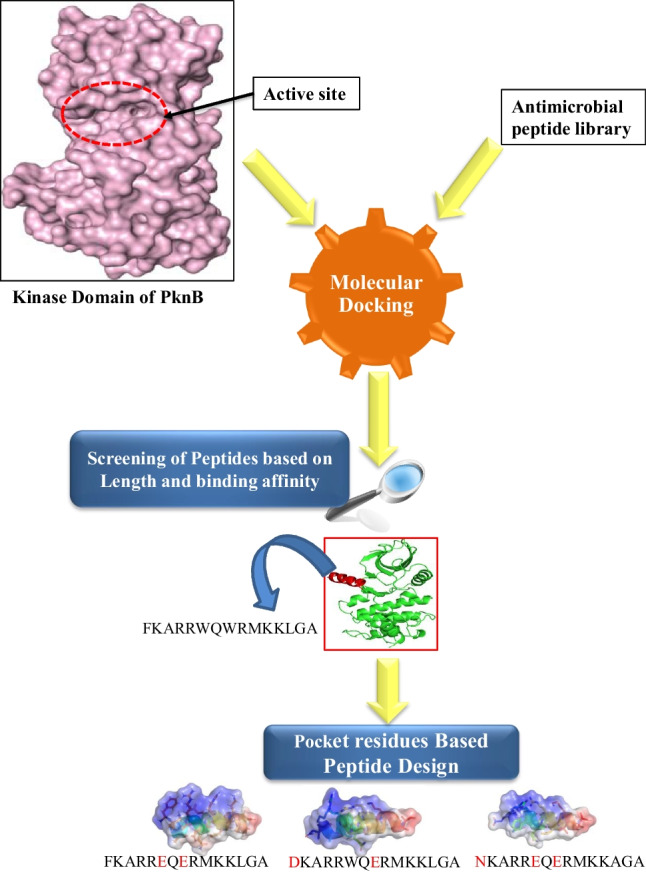

**Supplementary Information:**

The online version contains supplementary material available at 10.1007/s10930-024-10218-9.

## Introduction

The global threat of Mycobacterium tuberculosis (Mtb) is so significant that it has been included as one of the targets of the United Nations Sustainable Development Goals (SDGs) [1]. This superbug is powerful in its ability to colonise and resist most antibiotics. Therefore, the World Health Organization (WHO) has initiated efforts to eradicate Tuberculosis (TB) by 2030, and researchers worldwide are exploring all possible mechanisms to achieve this goal. One of the primary targets for inhibition is the mechanism of cell division of Mtb [2]. Deciphering the crucial proteins involved in this event and targeting them with small molecules has shown promising results. The current study aims to explore an alternative approach to slight molecule inhibition with peptide-based drugs [3].

**Fig. 1:**
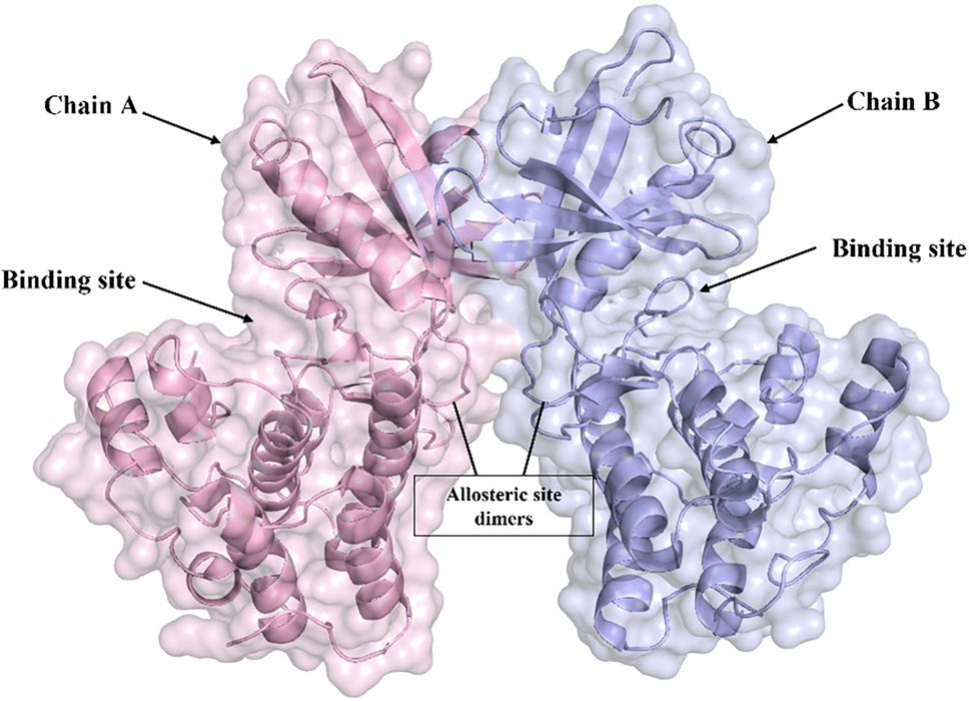
Surface view of PknB dimerisation as represented in PDB: 1MRU. The chains are homo-dimers, each with two lobes; the upper part is the N-terminus, while the lower part is the C-terminus. The loop part of both chains initiates the dimerisation process, allosterically attaching the two chains in a back-to-back orientation. The cleft part is the phosphate binding site

Protein Kinase B (PknB) is one of the 11 Serine/Threonine protein kinases (STPK) related to the metabolism of cell wall synthesis and cell division [4]. When an external component binds to the protein’s extracellular domain, PknB is activated, becoming a dimeric form of protein. Two similar dimeric proteins (4 chains) come closer and transduce the signal downstream by autophosphorylation [5]. Each chain of the protein complex contains an allosteric site responsible for the dimerization and a phosphorylating site [6]. Various small molecule inhibitors such as Imatinib, Clofazimine, Pyrazinamide, oxadiazoles, thiazolidines, and benzimidazoles bind to the ATP binding site to prevent the protein from functioning normally [7]. The protein has a dimeric form consisting of two chains oriented back-to-back, as shown in a co-crystal structure (PDB: 1MRU) [8]. Each chain has swollen amino (N) and carboxy (C) terminals separated by a hinge region, which serves as the binding site for an ATP analogue (refer to Fig. 1). The N-terminal lobe comprises β-sheets with a single long α-helix, while the C-terminal comprises only α-helices. When the two halves come together, they activate the kinase domain through an allosteric mechanism involving residues from Ala65 to Val74. The activity of one domain affects the other’s activity, leading to PknB auto-phosphorylation, followed by the phosphorylation of target proteins.

Inhibiting PknB activity can disrupt critical cellular processes, ultimately leading to the bacterium’s demise. The study aims to complement the active residues, namely Leu17, Lys40, Lys140, and Asp156, and their corresponding allosteric site to challenge the transduction of the downstream signal.

Peptide-based drugs are emerging as an innovative therapeutic modality due to their low potential for causing side effects. The FDA has recently approved peptide-based medicines for treating various diseases, including oncology, endocrinology, infectious diseases, cardiovascular diseases, and autoimmune disorders. Examples of these drugs include insulin analogues for diabetes management (such as insulin lispro and insulin glargine), peptide hormones for growth disorders (such as somatropin), peptide-based medicines for cancer therapy (such as leuprolide and octreotide), and peptide-based biologics for autoimmune diseases (such as adalimumab and ustekinumab) [9]. Antimicrobial peptides are particularly advantageous due to their widespread presence among different classes of Chordates. Short peptides are fast and efficiently diffusible, making them ideal for modern medical techniques. These peptides are naturally formed by the epithelial cells of the respiratory, gastrointestinal, and genitourinary tracts in mammalian hosts. Therefore, peptide-based drug therapy may be seen as a promising alternative mode of inhibitors from an immunological acceptance perspective. The advantages of a fully approved peptide drug may enhance the ease of penetration to cell membrane, increase specificity to the target and decrement of immunogenicity. Peptides can mimic natural hormones, enzymes, or other signalling molecules, making them applicable to a wide range of diseases.

Besides reckoning the peptide drugs, it is necessary to be cautious about any of the undesired random mutations of the candidate drug molecule and the target. This is where machine learning (ML) and deep learning (DL) render their applicability. ML models can analyze existing data on peptides and their properties (binding affinity, bioactivity, etc.) to predict these properties for new candidate peptides. This helps researchers prioritize the most promising candidates for further exploration [10]. ML can be used to virtually screen large libraries of peptides for those with desired interactions with target molecules. This can significantly reduce the time and resources needed for traditional wet-lab experiments [11]. Deep learning models can be trained on vast datasets of known peptides to design new sequences with specific functionalities. As instance we mention DeepImmuno-GAN [12], codon-based genetic algorithm (CB-GA) [13], PepVAE [14], ProteinGAN [15], HydrAMP [16], PepGAN [17], and peptide VAE [18].

While ML and DL offer significant advantages, there are limitations. One challenge is the availability of high-quality data for training models. Additionally, interpreting the predictions made by these models can be difficult.

Thus, before utilizing the above-mentioned models, we intend to employ a comprehensive approach to create a library of well-defined antimicrobial peptides to ensure their effectiveness. This involved multi-level computational methods such as protein-peptide docking, 100 ns molecular dynamics simulations, and binding free energy calculations. Notably, molecular mechanics with generalized Born and surface area solvation (MM-GBSA) was used to estimate binding free energy for protein-ligand complexes, and the energy values obtained for the identified lead peptide were compared to the Apo state of the protein.(M G et al. 2022; Ghosh et al. [19] The results showed that the length of the peptide and its combination with the protein were critical factors in effective conjugation to the binding site. Neither short nor long peptides were effective, and an intermediate length was needed for optimal results.

## Material and Method

### Selection and Preparation of Target Protein

The atomic crystal structure identified by PDB ID 1MRU was obtained from Protein Data Bank (PDB). It is a structure resolved through X-ray diffraction techniques with a resolution of 3 Å. The coordinate file included two chains with a bound phosphothiophosphoric acid-adenylate ester and two Magnesium ions. The retrieved protein file was prepared using PyMol version 2.3.4 [20] and UCSF Chimera version 1.6.1 [21]. The missing residue side chains were incorporated, and hydrogen atoms were added to the protein. A proper protonation of the amino acids and energy minimisation was then carried out using the molecular mechanics force field (AMBER FF) [22]. To continue, the prepared receptor file was formatted to pdbqt format using Obabel [23].

### Preparation of Peptide Library

In this study, we utilised the Antimicrobial Peptide Database (APD) to procure peptides and create a library [24]. The database consists of 3940 peptides, comprising 3146 natural antimicrobial peptides (AMPs) and 314 synthetic AMPs. Our goal was to supplement the protein’s hinge region, and as such, we restricted the length of the peptides generated between 5 and 25 amino acid residues. In the following descriptions, we will refer to the length of the peptides as ‘mers’, representing the number of constituent amino acid residues.

### Molecular Docking

There are three main categories of protein-peptide docking methods: template-based docking, local docking, and global docking techniques. Template-based docking is typically used when both the protein and peptide scaffolds are known, allowing for easy monitoring of interactions. Local docking, on the other hand, is limited by predefined binding site information. Global docking is used when there is no precise proximity of conformational details for the receptor or incoming peptide pose.

This analysis used a docking approach utilising binding site information with the receptor in three-dimensional conditions and a sequential list of peptides. We employed Autodock Crank Peptide (ADCP) [25], a molecular docking program that provides rigorous interaction and cluster-formed results 21. A grid box of 44 × 70 × 56 Å^3^ with a centre at 19.44, 27.91, and 40.55 was maintained to ensure accuracy. The validity of the grid information was confirmed by comparing the docked poses obtained from the co-crystallized ligand re-docked and PDB-driven complex.

### Interaction Analysis

The best complexes were chosen by sorting the peptides based on their binding affinity, as ranked in the log file. The peptides were initially divided into four groups based on their length: (a) 5 to 10 m, (b) 11 to 15 m, (c) 16 to 20 m, and (d) 21 to 25 m. The peptides were then sorted by their binding affinity in ascending order, and the selection was made from the peptides with the lowest numerical value. It was ensured that the selected peptide had no unfavourable pose against the receptor. The final complexes were visualised in PyMol. Five peptides were chosen from the varied length range based on their binding affinity.

### Molecular Dynamics

Each complex being studied contains two separate chains of amino acids. These chains likely undergo individual conformational changes under homeostatic conditions. A long-range, all-atom molecular dynamics simulation was conducted over 100ns to observe the interaction between chains and their effect on the length of the interacting peptide. The Gromacs platform, using the Charm36 forcefield, was employed to execute the simulation, with complexes being solvated in the TIP3P water model and truncated within an octahedron box [26]. A padding space of 10Å was maintained from the box walls. Before the simulation was run, the protein-ligand complex was equilibrated for 5ns in the NVT ensemble using Berendsen-thermostat at 310 K and then for 10ns in the NPT ensemble using the Parrinello-Rahman barostat algorithm at 1 bar. Post-simulation evaluations for spontaneity checks were carried out using Molecular Mechanics-Generalised Born Surface Area Solvation (MM-GBSA), with calculations being executed using the trajectory output files [27]. The Free Energy Landscape (FEL) was then calculated to estimate the protein’s folding behaviour against each screened molecule. This compares the molecular dynamic trajectory against the RMSD and the Radius of Gyration (ROG) to profile the free energy in a 3D plot.

### Design of Novel Peptides

**Fig. 2:**
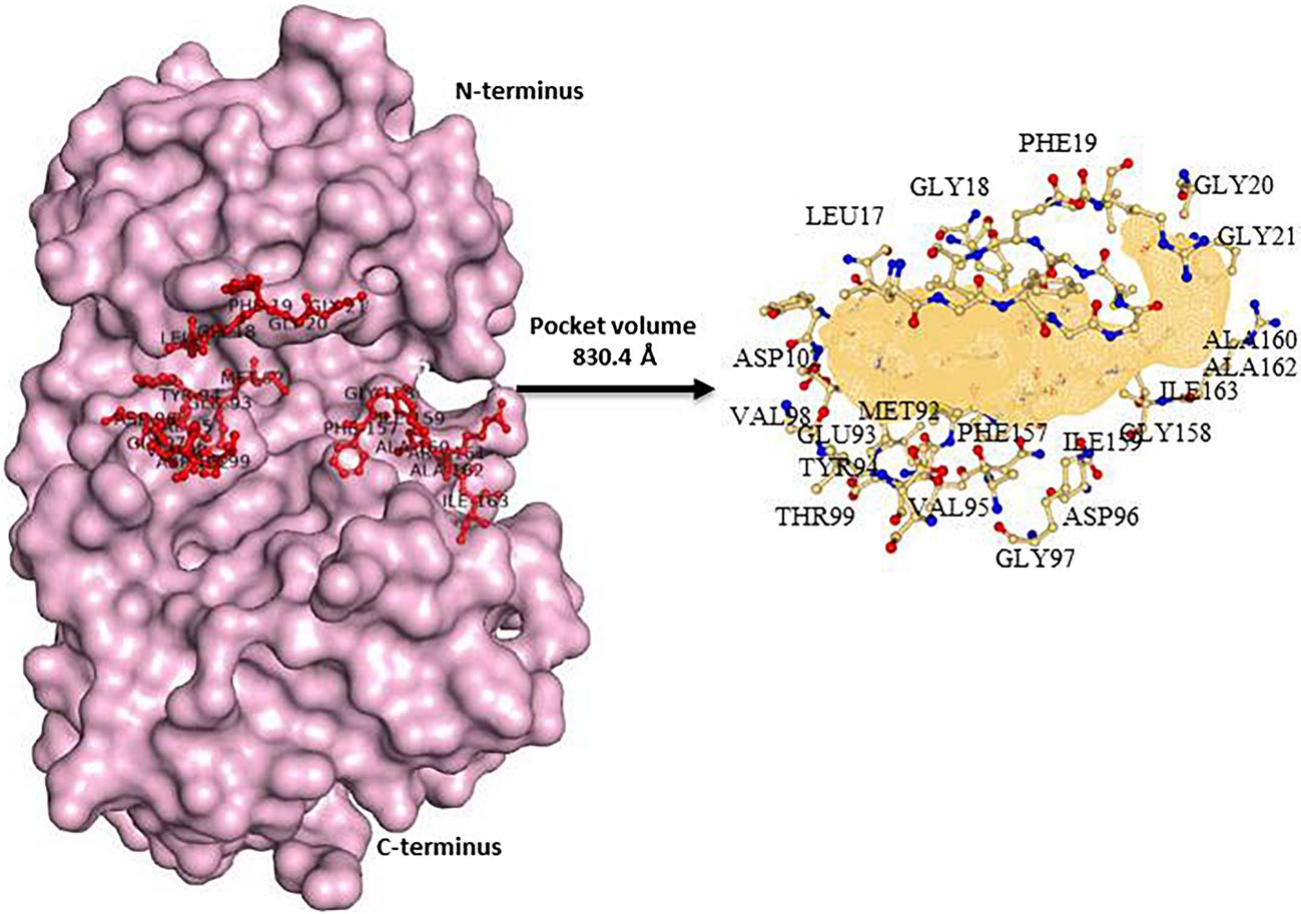
The surface view of the protein KD focuses on the hinge pocket, which is shown in mesh form with red sticks for the pocket-forming residues. The figure on the right is a graphical representation of the pocket topology (shown in mesh form) surrounded by pocket residues, shown in ball and stick form

Thus far, the peptides served as reference points for identifying suitable candidates for probe generation. Through the analysis of interchain interactions and pocket topology (as shown in Fig. 2), it became clear that modifying specific constituent residues in the screened peptides could enhance receptor-ligand coordination. Using ‘Proteins plus’ (https://proteins.plus/), we determined the volume and shape of the protein’s active site, while RING3 was used to judge inter-chain interactions [28]. To explore better substitutions, we submitted the complexes to GalaxyActDesign [29] Server, which replaced each residue with its 19 alternatives and created all possible peptide combinations. We then scored each newly formed peptide using a machine learning-based masked language model incorporating three-dimensional geometric features. Non-toxic peptides with better binding affinity were subjected to molecular docking and toxicity screening using the ToxIBTL server [30]. Finally, those peptides that demonstrated the most favourable results were considered for a second round of molecular dynamic simulation.

### Intrinsic Dynamics Analysis Using Elastic Network Models (ENM)

**Fig. 3:**
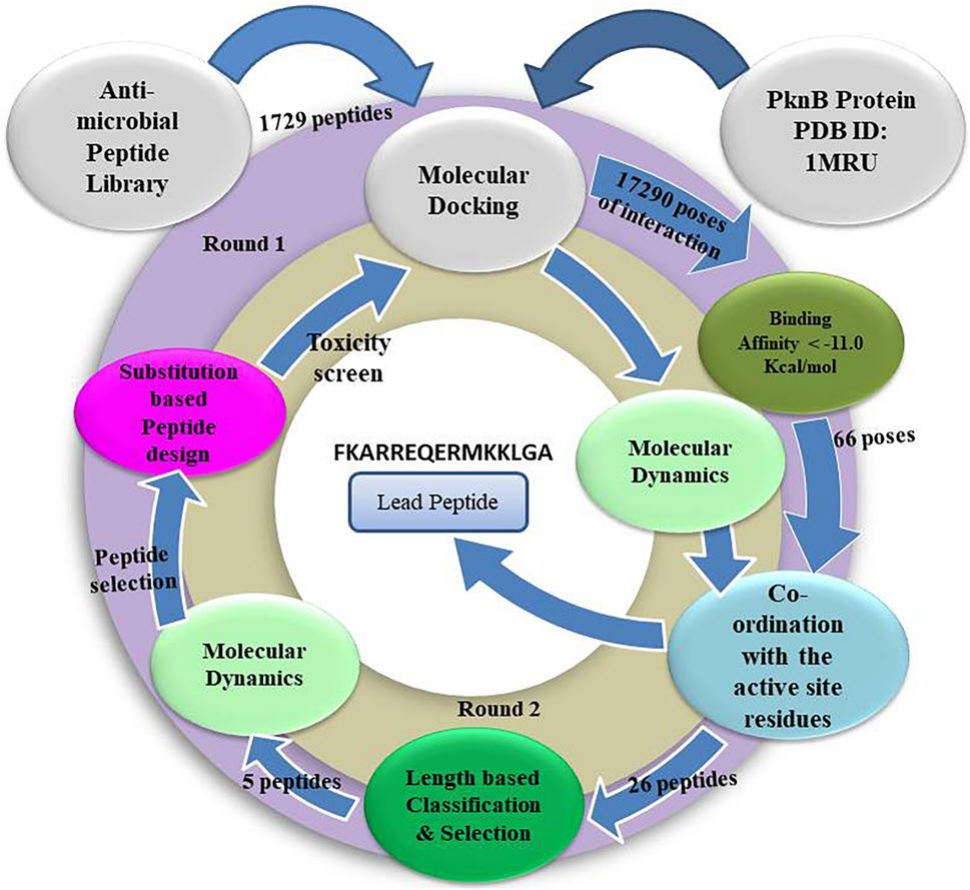
Workflow of Kinase Inhibiting Peptide Design using Recurring Interaction Analysis of antimicrobial peptide against PknB

The three-dimensional structural files were analysed using Elastic Network Models (ENM)(Park et al. [31] to achieve a conclusive state of peptide selection and incorporation. In the Elastic Network Analysis, the protein is represented by an ensemble of point-like particles connected by linear Hookean spring constants. This Coarse-Grained analysis gives a slow structural dynamic of inter-connected segments and helps to understand the mechanical behaviour of the macromolecules. ENM is often calculated using the Gaussian Network Model (GNM) and the Anisotropic Network Model (ANM). For a complex or apo-protein, the amino acids are represented by their α carbons as the point particles and are linked based on a cut-off distance among their coordinates. In the present study, the ENM is calculated using DynOmics [32]. Where the default cut-off distance is 7.3 Å, and the spring constant is kept at 1 Å (default value). During the calculations, the fluctuations of total particles (N) were estimated using a N x N connectivity matrix (the Kirchhoff matrix), where only the magnitudes were considered. Three-dimensional displacement factors were added to add directional information to this matrix. This new 3 N x 3 N matrix of the Anisotropic Network Model (ANM) provides the residual fluctuations corresponding to the reference structure. Incorporation of ENM would comprehend the effect of the referral peptide and design peptide to the apo-protein targeted for the study (Fig. 3).

## Results

### Protein Structure Optimisation

The protein kinase PknB, the receptor of our interest, belongs to the Ser/Thr family and consists of an N-lobe and a smaller C-lobe connected by a hinge region. We obtained the crystallographic coordinate file with the PDB ID 1MRU from the RCSB, which contained two homomers bound to a phosphothiophosphoric acid-adenylate ester and two magnesium ions. PLIP (Protein-Ligand Interaction Profiler) was used to determine the interacting amino acid residues, which are Leu17, Lys40, Kys140, and Asp15629. The targeted cleft of the protein is formed mainly by the following residues: Leu17, Gly18, Phe19, Gly20, Gly21, Met92, Glu93, Tyr94, Val95, Asp96, Gly97, Val98, Thr99, Asp102, Phe157, Gly158, Ile159, Ala160, Ala162, and Ile163. These residues are highlighted in red in the surface image shown in Fig. 2.

We prepared the retrieved protein crystallographic structure using PyMol version 2.3.4 and UCSF Chimera version 1.6.1. During the protein preparation step, all missing residue side chains in the structure file and hydrogen atoms added to the selected protein in the system were included. Finally, the protein structure was energy-minimized using the molecular mechanics force field (AMBER FF). The prepared receptor was then formatted to pdbqt format using Obabel.

### Library Generation

The most crucial aspect of this study on peptide design was the possession of antimicrobial properties. The University of Nebraska’s “APD3 Antimicrobial peptide database” has fulfilled this requirement for the past two decades (https://aps.unmc.edu). This comprehensive repository contains peptides (AMPs) from various sources, with 5626 entries of natural, synthetic, and predicted peptides. This vast amount of data was repeatedly filtered to remove duplicate entries and unspecified residual content to ensure accuracy. The filtered peptides were then selected based on their length, with only those between 5 and 25 residues chosen for further consideration. A total of 1729 peptides were thus selected and compiled into a ‘csv’ formatted file, each sequence prefaced with a user-defined sequence ID.

### Molecular Docking Experiment

The Autodock CrankPep Suite was utilised to perform the docking step. The program performs the docking procedure for each complex a minimum of 1000 times and clusters the poses based on population size. These arrangements produce various possible poses of the peptide against the receptor molecule. The top ten poses were ranked and enlisted to streamline the output for each peptide. Consequently, the number of poses increases to a multiple of ten based on the given input peptide sequence of 1729. To sort this extensive data, all individual ranking values were initially sorted. Two scenarios are expected to occur, where different peptides may have similar binding affinity, and the same peptide might have a different pose corresponding to a wide range of binding affinity.

The highest binding affinity recorded was − 16 kcal/mol. A binding affinity cut-off of <= -11 kcal/mol was considered to maintain a systematic selection of peptides, resulting in 66 chosen peptide poses. If any of these peptides had a pose corresponding to a positive value in the initially derived result, all results for that particular peptide were discarded. The screened peptides were then examined for their association with the binding site cleft using PyMol. Finally, 26 peptides were discovered to have better binding affinity besides interacting with active site residues. Peptides, including their sequence ID and the coordinated residues of the receptor, are provided in SI1.

### Interaction Analysis

The peptides with higher binding affinities were classified into four groups. All the 26 protein-peptides complexes were observed using LigPlot. The significant interchain interactions are tabulated in Table 1. Five complexes with better results are reported elaborately in SI2. It was found that even for the varied lengths of incoming peptides, a few residues behaved similarly. The residues viz.; Glu15, Tyr94, Asp102 were seen to commonly form H-bonds with the peptides invariably. However, with the increment of the peptide, a few more coordinations were added up. Besides the H-bond, Asp96, Asp102, and Arg101 formed a salt bridge to the peptide fragments.

**Table 1:** Interacting residues of the receptor to the peptides with high binding affinity

Sequence Id (number of amino acid residues in the peptide)	Binding affinity (Kcal/mol)	Hydrophobic Interaction	Other Interaction
SEQ1516 (06)	-14.4	Ile16, Leu17, Gly18, Val25, Val95, Val98, Thr99, Met145	Glu15, Tyr94, Asp96, Gly97, Asp102 (Hbonds)
SEQ785 (11)	-12.1	Glu15, Ile16, Leu17, Gly18, Val98, Thr99, Thr106	Tyr94, Asp96, Asp102 (Hbonds)
SEQ578 (15)	-15.7	Ile16, Gly18, Phe19, Val98, Ile103.	Glu15, Leu17, Tyr94, Asp96, Gly97, Asp102, Glu107 (H Bond), Asp96, Asp102 (Salt Bridge).
SEQ1603 (18)	-12.2		Leu17, Phe19, Srg101, Asp102, His105, Thr106 (H Bond), Arg101, Asp102 (Salt Bridge)
SEQ488 (24)	-12.6	Gly18, Phe19, Arg101, Asp102, His105, Thr106, Lys140, Ala142, Thr164, Ala165, Tyr167, Ser205, Pro206	Glu15, Ile16, Leu17, Ile163, Gly203 (Hbonds)
SEQ578(substituted) W6E and W8E	-13.0	Leu17, Tyr94, Val95, Gly97, Val98, Asp102, Thr106, Glu107, Met145	Tyr94, Gly97, Asp102, Thr106(Hbonds)

### Molecular Dynamics

Selected docked complexes were subjected to molecular dynamics (MD) simulations to understand the conformational changes and identify critical residues that govern their activity. The atomic movements of the complexes were observed for a period of 100 ns, and the frames generated per picosecond were analysed to measure their positional displacements (RMSD), the compactness of the receptor (ROG), inter-chain Hydrogen bonds (H-Bonds), and the thermodynamic favorability of complex formation using MMGBSA, FEL, and PCA. The results were then compared to the Apo-protein.

**Table 2:** Parameters of post-molecular dynamic analysis

Parameter		APO	S1516 (6aa)	S785 (11aa)	S578 (15aa)	S1603 (18aa)	S488 (24aa)	P578 (15aa)
Receptor RMSD	AVG	3.18	3.92	2.87	3.00	3.16	2.99	3.47
	MIN	0.01	0.01	0.00	0.00	0.00	0.00	0.00
	MAX	4.69	5.84	4.45	5.01	5.49	3.75	4.66
Peptide RMSD	AVG	-	4.66	3.16	3.90	6.82	3.61	4.44
	MIN	-	0.00	0.00	0.00	0.00	0.01	0.01
	MAX	-	6.20	4.50	5.76	8.43	5.46	5.70
Receptor RMSF	AVG	1.74	1.79	1.47	1.41	1.90	1.41	1.57
	MIN	0.65	0.67	0.52	0.56	0.67	0.52	0.61
	MAX	9.18	9.14	6.46	8.17	7.68	5.18	7.03
ROG	AVG	20.66	20.68	19.99	20.29	20.12	20.17	21.36
	MIN	19.51	19.54	19.48	19.53	19.34	19.52	19.58
	MAX	21.43	21.81	21.11	21.42	21.67	20.77	27.50

#### Protein RMSD

The impact of the peptides on the targeted protein can be observed through the root mean square deviation (RMSD) of the receptor backbone (refer to Table 2). However, it’s important to note that the effect is not directly proportional to the length of the peptides. The shortest peptide (S1516 with six aa) shows more significant residual displacements. This trend is also observed in the latter half of the trajectory for the 18 amino acid length peptides (S1603) (as shown in the comparable figure in SI3A). Interestingly, the RMSD values decrease compared to the apo state of the protein when an intermediate peptide length is incorporated for interaction. We will discuss the impact of the substituted form of S578, i.e., P578, in more detail in the discussion section.

#### Peptide RMSD

The RMSD value of a receptor peptide does not show a linear relationship with the peptide’s length. This is because the peptide can be accommodated in the active site region, allowing for the movement of the active site residues. The discussion section further elaborates on observing active site residue movement during dynamic studies.

#### RMSF of the Receptor

The residual role in maintaining the coordination and function of the protein can be attributed to the individual fluctuations of the receptor’s residues. Notably, the RoG plots (accessible in the SI3C) indicate that the residues surrounding the cleft area exhibit higher peaks, indicating more motion than the pocket residues. Moreover, it appears that the protein experiences fewer fluctuations when in contact with the peptide than in its unbound form (apoprotein).

#### Radius of Gyration

The radius of gyration (ROG) reveals the size of the complexes, typically around 20 Å with a range of ± 1 Å. Interestingly, compared to the Apo-protein (black), all complexes except S1516 (green plot) appear to contract slightly, suggesting a potential unfolding of the receptor during interaction with the shortest peptide. In contrast, S785 (cyan) with 11 amino acids exhibits a consistently folded state. The numerical values can be cross-checked against Table 2, and a corresponding plot is accessible in the supplementary information (SI3B).

## Discussion

**Fig. 4:**
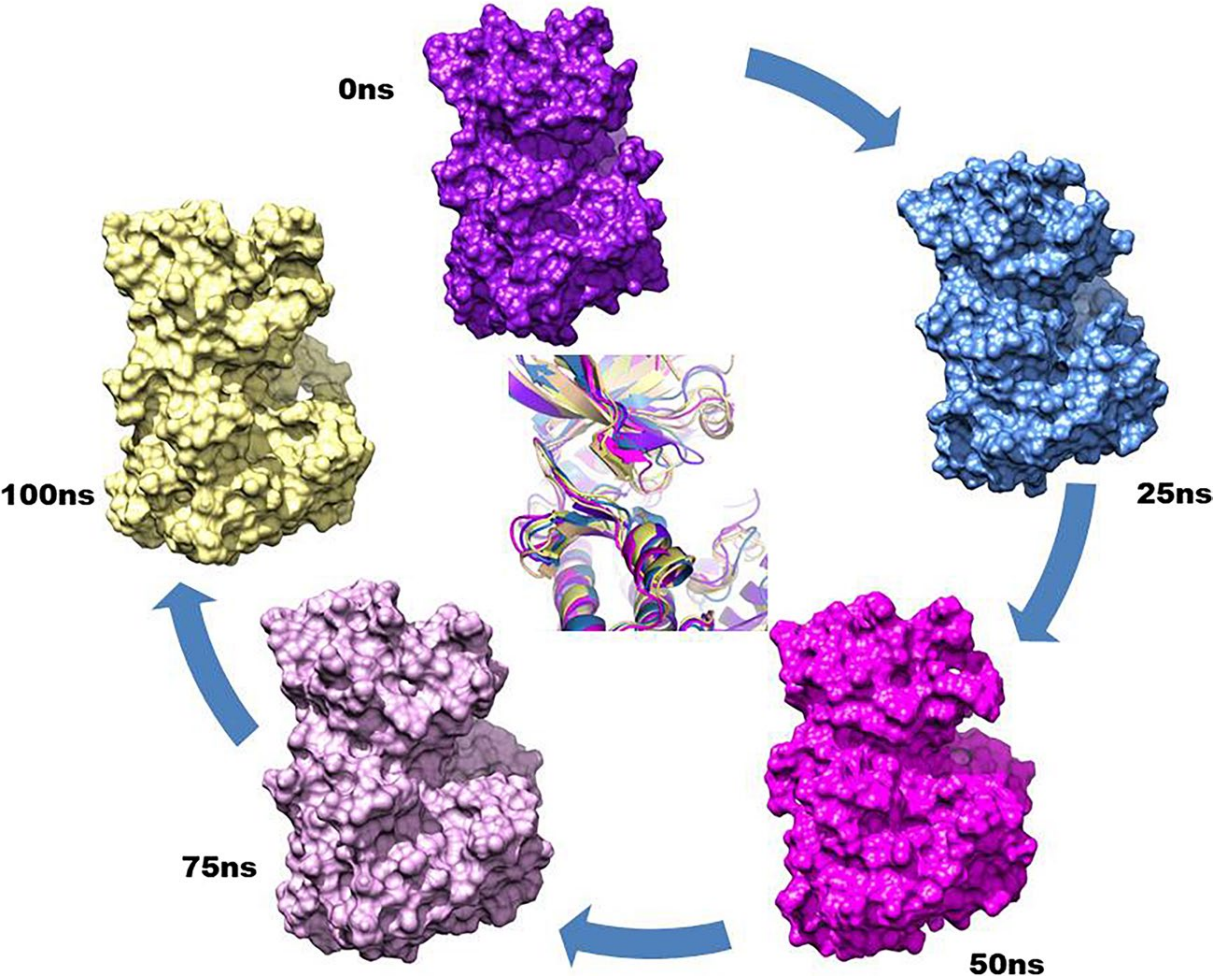
Topological changes of the active site of the PknB protein (Apo-form) is observed for 100ns. The frames are differentially coloured with respect to time to compare the displacements in the secondary structure in the centre. A noteworthy change in the short β-strand, above the catalytic site and the α-helix below, is seen

The comparative analysis of the peptides varying in length shed light on the fact that mere length is not the decisive factor in generating a peptide probe. In the dynamic study of the apo-protein, it is seen that the area of active pocket fluctuated variably. Although the volume of pocket the was 830.4 Å, the two regions, namely the beta-strand forming Leu17, Gly18, Phe19, Gly20 and Gly21, and the alpha helix forming residues Leu100, Arg101, Asp102, Ile103, Val104, His105, Thr106 and Glu107 were in continuous movements. Figure 4 shows that during the tenure of 100 ns, the pocket area has skewed from its original shape. The area beyond the active site was also seen to be opened up. Thus, rather than considering an antimicrobial peptide of a different length, selecting a combination of amino acids is more crucial. Hence, from the first round of peptide selection, the approximate length of peptides was decided, and later, these peptides were substituted with the constituent’s amino acid that could complement the charge of the active site pocket residues.

### Design of New Peptides

The referral peptides from the initial screening were processed using the Galaxy webserver to create new peptides with substituted amino acids. These resultant peptides were evaluated based on their score, which typically corresponded with increased affinity. In our study, scores ranged from 5 to -7, and our goal was to enhance the complementary charge of residues that conceal the receptor pocket. We only selected higher-value substitutions, limiting our score threshold to 3. Using point substitutions, we generated multiple peptides for each of our four original peptides. Toxicity screening was conducted on each set of peptides using ToxIBTL, a tool for predicting peptide toxicity based on information bottleneck and transfer learning. The peptides that passed toxicity screening were then subjected to peptide docking using the ADCP tool. Ultimately, we selected inhibiting peptides with poses corresponding to higher binding affinity against the pocket [33].

**Fig. 5:**
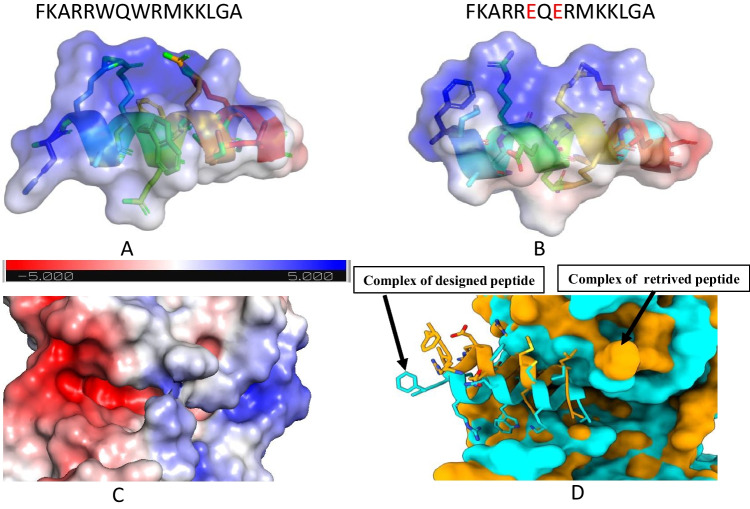
Surface view concerning the charge density is represented from A to C. The colour intensity differs from red (highly –ve) to faded (neutral)and extends to blue (highly +ve) charge. The charge density variation was observed using the APBS plugin in PyMol. **A** The original peptide S578 of 15 amino acid (**B**) is the point-stimulated peptide at the 6th and 8th positions. **C** The pocket forms the cleft region of the protein (**D**) superimposition of the wild and the substituted complex. The Orange represents the wild type, and the Cyan indicates the substituted docked complex. The side chains of the peptides are shown as sticks protruding out of their cartoon-formed backbones

Below, we present the substitution carried out to S578. The new peptide formed schematically by substituting the tryptophan(W) at the 6th and 8th positions with the Glutamate (E) alters the topology of the pocket-forming region (Fig. 5).

The receptor’s acceptance of the peptides was tested using MMGBSA and FEL calculations. The binding free energy (ΔG) value changed from − 7.76 (kcal/mol) to -13.65 (Kcal/mol) after the substitution. The MMGBSA values are submitted in the SI4. The FEL converged equally, but the substituted peptide’s complex covered more residues to decompose (data provided in SI6-9). The Principal Component Analysis (PCA) indicates intermediate combinations of components that affect the constituent molecules with similar behaviour, which are grouped in clusters (data provided in SI5). In the substituted peptide complex, there are fewer clusters but with a larger size than that of the wild type. Notably, peptides that tend to remain intact with the active pockets had numerically higher binding free values than those bound to an untargeted site of the protein. Two inferences can be drawn from the numerical figures and the molecular dynamics trajectories. Firstly, like the receptor, peptides act as a rigid body with lesser torsion than small molecules. Peptides of shorter lengths get more opportunities to engage themselves other than the active site. Secondly, the solvent plays a crucial role in competing with the peptides when the active sites are more exposed.

### Intrinsic Dynamics Analysis Using Elastic Network Models (ENM)

The comparative residual cross-correlation deciphers the synchronistic residual fluctuations of the protein and illustrates the impact of the incorporated peptide. In the Elastic Network Model (ENM) the nodes are defined by the Cα-atom coordinates, and the probable bonded and non-bonded among the pairs of residues are considered as the springs. The force of the spring γ, depends on a cut-off distance (rc). Usually, rc is taken to be 7.0 Å, based on the radius of the first coordination shell. For one node (say i) at equilibration if the position is defined by $\:{R}_{i}^{0}$ and the instantaneous position by R_i_. The fluctuations can be defined by.

$$\triangle{\mathrm R}_{\mathrm i}={\mathrm R}_i-\mathrm R_i^0$$

Similarly, vector of the displacements between two residues i and j are stated as

$$\triangle R_{ij}=R_{ij}-R_{ij}^0$$

Or

$$\triangle{\mathrm R}_{\mathrm{ij}}=\triangle{\mathrm R}_{\mathrm j}-\triangle{\mathrm R}_{\mathrm i}$$

For a fluctuation in Gaussian Model (GNM) of N number of nodes, the potential V_GNM_ is defined by ${\mathrm V}_{\mathrm{GNM}}=\frac\gamma2\left[{\textstyle\sum_{\mathrm i,\mathrm j}^N}\;\Gamma ij\lbrack\left(\triangle{\mathrm X}_{\mathrm i}-\triangle{\mathrm X}_{\mathrm j}\right)^2+\left(\triangle{\mathrm Y}_{\mathrm i}-\triangle{\mathrm Y}_{\mathrm j}\right)^2+\left(\triangle{\mathrm Z}_{\mathrm i}-\triangle{\mathrm Z}_{\mathrm j}\right)^2\right]$

Here X, Y and Z are the components of the fluctuation vector ΔR_i_. The state vector gives the system (N) the difference between the referral state (user input complex) and the equilibrium state. In an N-by-N topology matrix, the connectivity of a pair of nodes (n) is stated as Γ_ij_ =-1, where ‘i’ is the subjected node and ‘j’ corresponds to the connected node. Rouse stated the Matrix of this connectivity as a Hessian matrix (H)30.

Thus, in the matrix $\mathrm\Gamma^{-1}=\mathrm C$

**Fig. 6:**
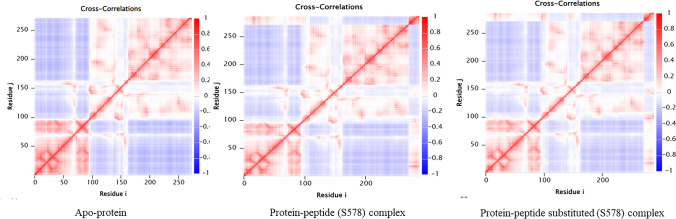
Cross-correlation matrices form by calculating the ENM

C is the cross-correlation matrix of the pair of residues, on a comparison of the cross-correlation matrix cited in Figure No. The scale gradually changes from anti-correlation (-1) to a perfectly correlated (1) pair. Summarily, in both bound and unbound states, the protein has correlations in three segments: (a) the N terminal initial segments from 0 to 100, (b) the intermediate region from 100 to 150 and (c) C terminal segments from 150 to 280, respectively. Upon incorporating the peptide, a correlation is seen between the protein and peptide at the N terminal. Concomitantly, a correlation (colour intensity) decrement is found in the hinge region. The molecular motion- animations (provided in SI) showed a better impact of the peptide on the protein. The possible inference drawn from this analysis is that a steric hindrance was felt among the pocket-forming residues. At the same time, the protein was perturbed with a 15 amino acid length peptide (Fig. 6).

## Conclusion

This study aimed to establish a systematic approach to designing peptides through a knowledge-based analytical method. This approach entails screening a list of potent antimicrobial peptides using a genetic algorithm-based docking procedure and simulation-based interaction behaviour. New peptides are generated and filtered for toxicity values by evaluating the charged complementarity. The 15 amino acid length modified peptide discussed in this study displayed the increment of co-ordinations to the receptor. Before the modification, the length-based selection helped us to scrutinise peptide candidates that could be induced in a lock-and-key manner. The interaction analysis procedures are repeated to verify the efficacy of the newly developed peptide-based inhibitors. This iterative protocol development analysis has demonstrated that the combination of the constituent residues must also be considered in addition to peptide length. The study hints at a new avenue from the immunology perspective of complement-based therapeutics developments. However, the study draws out the challenges of fine-tuned molecule development. Its reframing of the amino acid residue is currently marked only on the complementarity of the receptor’s pocket-forming residues. However, domain movement with respect to environmental and functional requirements and the entanglement of the induced peptide ought to be verified. Thus, invariable to the case study, we feel the development of ML and DL algorithms considering the pitfalls of similar studies.

### Supplementary Material

Supplementary Material 1.

## Data Availability

No datasets were generated or analysed during the current study.
