# Supplementary material for: Identification and Design of Novel Potential Antimicrobial Peptides Targeting Mycobacterial Protein Kinase PknB
Source: Protein J. 2024 Jul 16;43(4):858–68. doi: 10.1007/s10930-024-10218-9 (PMC11345320; doi:10.1007/s10930-024-10218-9)
Supplement: Supplementary file 1 — Supplementary Material 1. [file 10930_2024_10218_MOESM1_ESM.docx]

**Supplementary Information**

**Identification and Design of Novel Antimicrobial Peptides Targeting Mycobacterial Protein Kinase PknB**

Hemchandra Deka^1^, Atul Pawar^1^, Monishka Battula^2^, Ayman A. Ghfar^3^, Mohamed E. Assal^3^ Rupesh Chikhale^4^*

*^1^SilicoScientia Private Limited, Nagananda Commercial Complex, No. 07/3, 15/1, 18^th^ Main Road, Jayanagar 9^th^ Block, Bengaluru – 5600413*

*^2^Department of Bioinformatics, Rajiv Gandhi Institute of IT and Biotechnology, Bharati Vidyapeeth Deemed to be University, Pune-Satara Road, Pune, India*

*^3^Chemistry Department, College of Science, King Saud University, Riyadh 11451, Saudi Arabia*

*^4^Department of Pharmaceutical and Biological Chemistry, School of Pharmacy, University College London, London, UK.*

**Table of Contents**

| Serial Number | Topic | Page Number |
| --- | --- | --- |
| SI 1 | Interacting residues of the receptors to the peptides with high binding affinity | 2 |
| SI 2 | 2D interaction of the peptides with the pocket residue(Ligplot) | 4 |
| SI 3 | Post Molecular Dynamics plots of the complexes formed by the selected peptides and the Apo proteins | 5 |
| SI 4 | Energy profile of the MMGBSA calculations of the Apo protein and the peptide complexes | 6 |
| SI5 | PCA of Complexes formed by the selected peptides and Apo protein | 7 |
| SI 6 | Free Energy Landscape (FEL) of Complexes formed by the selected peptides and Apo protein | 6 |
| SI 7 | Post dynamic plots of the complexes formed by the substituted peptides | 9 |
| SI 8 | PCA of Complexes formed by the substituted peptides | 10 |
| SI 9 | Free Energy Landscape (FEL) of Complexes formed by the substituted peptides | 11 |

**S1 Interacting residues of the receptors to the peptides with high binding affinity**

Table S1 Interaction residues of the receptors to the peptides with high binding affinity

| **Molecules ID** | **Binding Affinity (kcal/mol)** | **Hydrophobic Interaction** | **Other Interaction** |
| --- | --- | --- | --- |
| SEQ1516 | -14.4 | Ile16, Leu17, Gly18, Val25, Val95, Val98, Thr99, Met145 | Glu15, Tyr94, Asp96, Gly97, Asp102 (Hbonds) |
| SEQ1528 | -11.2 | Gly97, Val98, His105, Thr106, Glu107 | Glu15 (Hbonds) , Ile16, Leu17, Asp96,Asp102 |
| SEQ0648 | -11.9 | Glu15, Ile16, Leu17, Gly18, Phe19, Tyr94, Val95, Gly97, Val98, Thr99, Asp102, Ile103, Glu107, Met145 | Asp96, Thr106, Ala142 (Hbonds) |
| SEQ0649 | -11.6 | Glu15, Ile16, Gly18, Phe19, Asp96, Gly97, Val98, Met145 | Leu17, Asp102, His105, Thr106 (Hbonds) |
| SEQ0770 | -11.5 | Arg57, Arg58, Asn62, Asn67, Tyr75, Asp76, | Leu66, Ala64, Gln61 (Hbonds) |
| SEQ0737 | -11.2 | Phe19, Gly20, Gly21, Lys140, Ala142, Ile163, Thr164, Ala165, Gln166, Tyr167, Pro206, Val209, | Arg101, Asn143, Asp156, Arg61 (Hbonds) |
| SEQ0738 | -11.5 | Pro69, Ala70, Arg114, Ala151, Val152 | Glu117, Asp121, Gln124, Asn150, Arg255 (Hbonds) |
| SEQ0785 | -12.1 | Glu15, Ile16, Leu17, Gly18, Val98, Thr99, Thr106 | Tyr94, Asp96, Asp102 (Hbonds) |
| SEQ0787 | -11.2 | Arg35, Asp36, Val37, Tyr94, Val95, Asp96, Ser147, Ala148 | Thr149,Tyr75, Glu93 (Hbonds) |
| SEQ0654 | -11.3 | Val98, Gly97, Asp102, Thr99, Thr106, Phe19, Leu17, Gly18, Ile16, Asp96 | Glu15 |
| SEQ0653 | -15.3 | Leu17,Gly18, Val98, Met145 | Asp102, Gly97, Glu15 (Hbonds) |
| SEQ0578 | -15.7 | Ile16, Gly18, Phe19, Val98, Ile103. | Glu15, Leu17, Tyr94, Asp96, Gly97, Asp102, Glu107 (H Bond), Asp96, Asp102 (Salt Bridge). |
| SEQ813 | -12 | Leu17, Gly18, Leu27, Tyr94, Asp96, Gly97, Thr99, Met145, Val195, Val198 | Glu15, Ile16, Asp102, Thr106 (H Bond), Tyr94 (Salt Bridge). |
| SEQ588 | -14.7 | Ile16, Gly18, Phe19, Val98, Ile103 | Glu15, Leu17, Tyr94, Asp96, Gly97, Asp102, Glu107 (H Bond), Asp96, Asp102 (Salt Bridge) |
| SEQ0632 | -13 | Ile16, Leu17, Gly18, Phe19, Gly21, Arg43, His105, Thr106, Ala162, Ile163, Thr164, Ala165 | Arg101, Asp102, Arg161, Gln166 (H Bond) |
| SEQ0882 | -11.3 | Arg35, Ser147, Ala148, Lys153, Val195. | Asp36, Tyr75, Glu93, Tyr94, Asp96, Thr149 (H Bond), Glu93 (Salt Bridge). |
| SEQ1603 | -12.2 |  | LEU17, PHE19, SRG101, ASP102, HIS105, THR106 (H Bond), ARG101, ASP102 (Salt Bridge) |
| SEQ1647 | -11.4 | Asp36, Arg35, Tyr75, Glu93, Ala151, Ser147, Ile71, Thr149, Ala148, His68, Asn67, Ala73, Lys153 | Tyr94, Asp96 (Hbonds) |
| SEQ0705 | -11 | His68, Gln124, Ala70, Asp121, Pro69, Gln131, Phe128, Thr149, Glu93, Arg255, |  |
| SEQ0052 | -11.4 | Ala173, Gly175, Val207, Ala210 | Arg174, Arg215, Asp176 (Hbonds) |
| SEQ0722 | -11.5 | Ile16, Phe19, Met1, Thr2 | Glu24, Glu15 (Hbonds) |
| SEQ0743 | -11 | Pro69, His68, Tyr79, Phe128, Lys153, Glu93, Arg35, Tyr94 | Asn67, Asp36, Asp96 (Hbonds) |
| SEQ0881 | -12 | Ile16, Gly20, Arg43, Gln166, Arg101, Gly21, Ala162, Gly18, Ala142, Phe19, Thr99 | Glu15, Asp102, Glu107, His105, Arg161, Ile163 (Hbonds) |
| SEQ0714 | -11.1 | Pro69, Glu93, Ala73, Ile71, Arg35, Tyr75, Val37, Lys153, Ala151, His68, Tyr94 | Asn67, Ala23, Asp36 (Hbonds) |
| SEQ0488  24 | -12.6 | Ser205, Ala165, Pro206, Lys140, Thr164, Ala142, Tyr167, Arg101, Asp102, His105, Phe19, Gly18, Thr106 | Gly203, Ile163, Leu17, Ile16, Glu15 (Hbonds) |
| SEQ1516 | -14.4 | Ile16(A), Leu17(A), Gly18(A), Val25(A), Val95(A), Val98(A), Thr99(A), Met145(A), | Glu15, Tyr94, Asp96, Gly97, Asp102 (Hbonds) |

***S2. 2D interaction of the peptides with the pocket residue(Ligplot)***


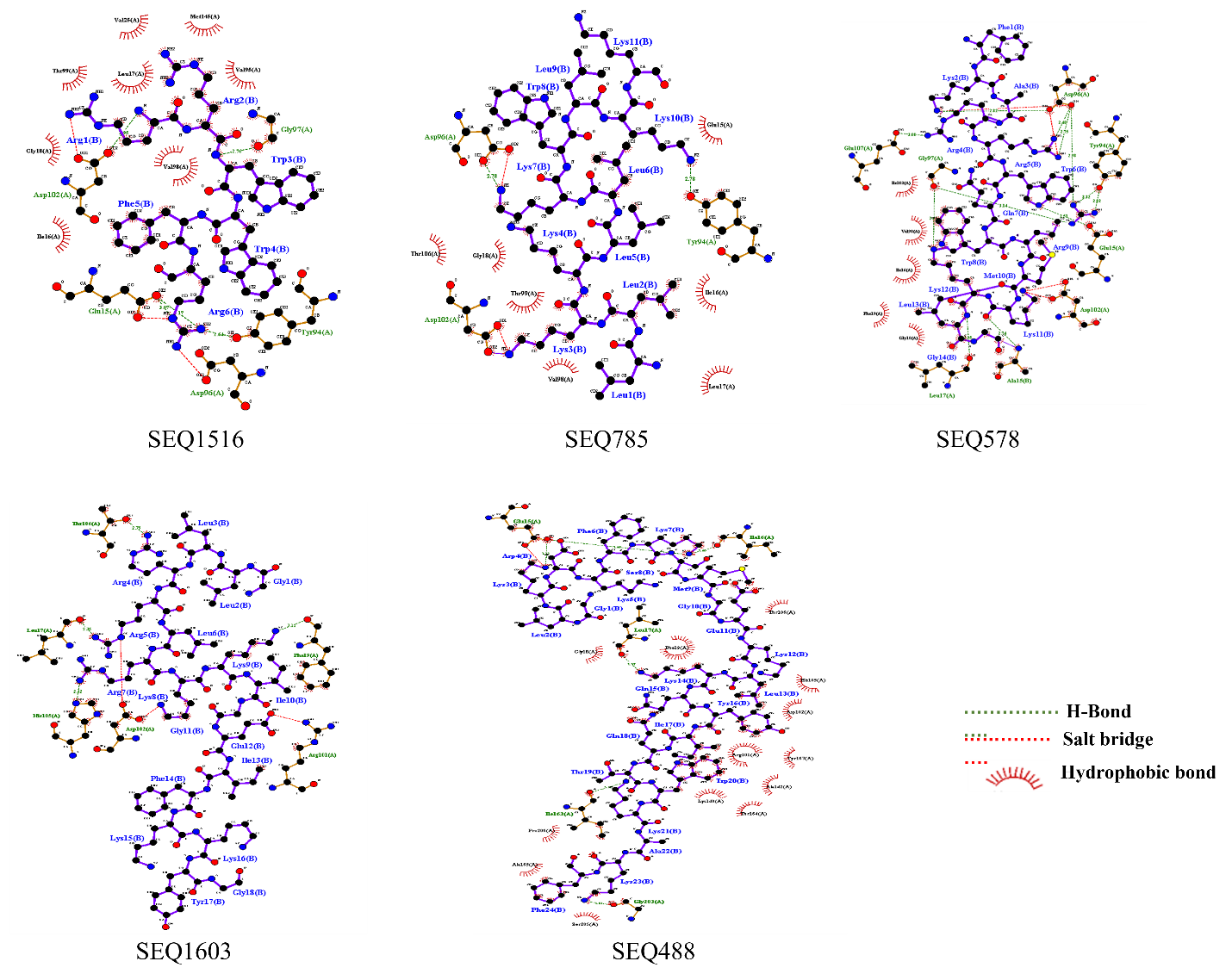


Fig S2 Interaction of peptides to the pocket residues represented in 2D form using LigPlot. The purple lines indicate bonds among the peptides and yellow lines indicates the bond of the pocket residues. H-bonds are represented by green lines. Red lines represent salt bridges and red dotted arc represents hydrophobic bonds.

***S3 Post Molecular Dynamics plots of the complexes formed by the selected peptides and the Apo proteins***

Fig S3 Post molecular dynamic analysis of the peptides A. protein RMSD, B. Radius of Gyration (RoG), C. Interchain hydrogen bonds and D. RMSF of the protein molecule.

**Interchain H-bonds**

The inter-chain H-bonds were not in a regular proportion with the length of the peptides. Despite being the longest peptide, S488 (red) with 24 amino, did not form a constant high number of H-bonds. There were only two notable instances, one around 10^th^ ns and another 85^th^ ns, about 8-9 bonds coordination was formed. S1603 (yellow) with 18 amino acids residues had over 5 H-bonds for a major tenure of the 100ns trajectory. It even formed 8-10 associations for several instances. While S578(purple)with its 15 amino acids irregular interactions, S785(cyan) with 11 residues formed a constant 3 h-bonds and more at irregular intervals. Interestingly, S1516 (green) having 6 amino acid residue formed 4-5 bonds at regular intervals in the first 50 ns and later increased to form several instances of 8-10 bonds. This indicated multiple posed association of the peptide.

***S4. Calculation of binding free energy through MMGBSA approach***

The Binding free energy checks the potentiality of peptide bound complexes. Using MMGBSA calculation over the trajectory values the binding free energy has been deduced. Lower the energy value, higher is the contribution of the energy components. The table (Table 3) clearly shows that the bonded components majorly effect the protein peptide interaction over the non-bonded components. Among the five peptides surprisingly the longest and the shortest peptides demonstrates a similar free energy. Whereas, intermediate

Table S4 Binding free energy of the protein peptide complex calculated using MMGBSA

| Energy Component | S1516 | S785 | S578 | S1603 | S488 | Substituted  578 |
| --- | --- | --- | --- | --- | --- | --- |
| ΔEEL (kcal/mol)) | -683.35 (±59.97) | -850.97 (±131.65) | -646.63 (±379.43) | -995.07 (±255.68) | -611.45 (±165.86) | -598.11  (±184.96) |
| ΔVDWAALS (kcal/mol)) | -26.41 (±5.44) | -23.65 (±8) | -12.74 (±12.82) | -22.13 (±9.62) | -54.86 (±26.43) | -21.46 (±9.67) |
| ΔEGB | 687.25  (±55.78) | 864.89  (±132.62) | 653.92 (±386.34) | 1002.14  (±256.92) | 643  (±172.83) | 610 (±189.33) |
| ΔESURF | -4.93  (±0.86) | -3.91  (±1.15) | -2.31  (± 2.31) | -4.28  (±1.72) | -7.77  (±3.67) | -4.19 (±1.85) |
| ΔGGAS | -709.76  (±61.19) | -874.62  (±136.16) | -659.37  (± 391.82) | -1017.20  (±262.65) | -666.31  (±178.53) | -619.57  (±193.3) |
| ΔGSOLV | 682.32  (±55.25) | 860.97  (±131.74) | 651.61 (±384.07) | 997.87  (±255.45) | 636.12  (±170.81) | 605.93  (±187.63) |
| ΔG (kcal/mol)) | -27.44 (±7.45) | -13.65 (±6.38) | -7.76 (±8.56) | -19.33 (±9.8) | -30.19 (±16.25) | -13.65  (7.69) |

***S5. PCA of Complexes formed by the selected peptides and Apo protein***

Fig S5 A) PCA plot of S785 B) PCA plot of S488 C) PCA plot of S1516 and D) PCA plot of S578 E) PCA plot of S1603 and F) PCA plot of Apoprotein

***S6 Free Energy Landscape (FEL) of Complexes formed by the selected peptides and Apo protein***


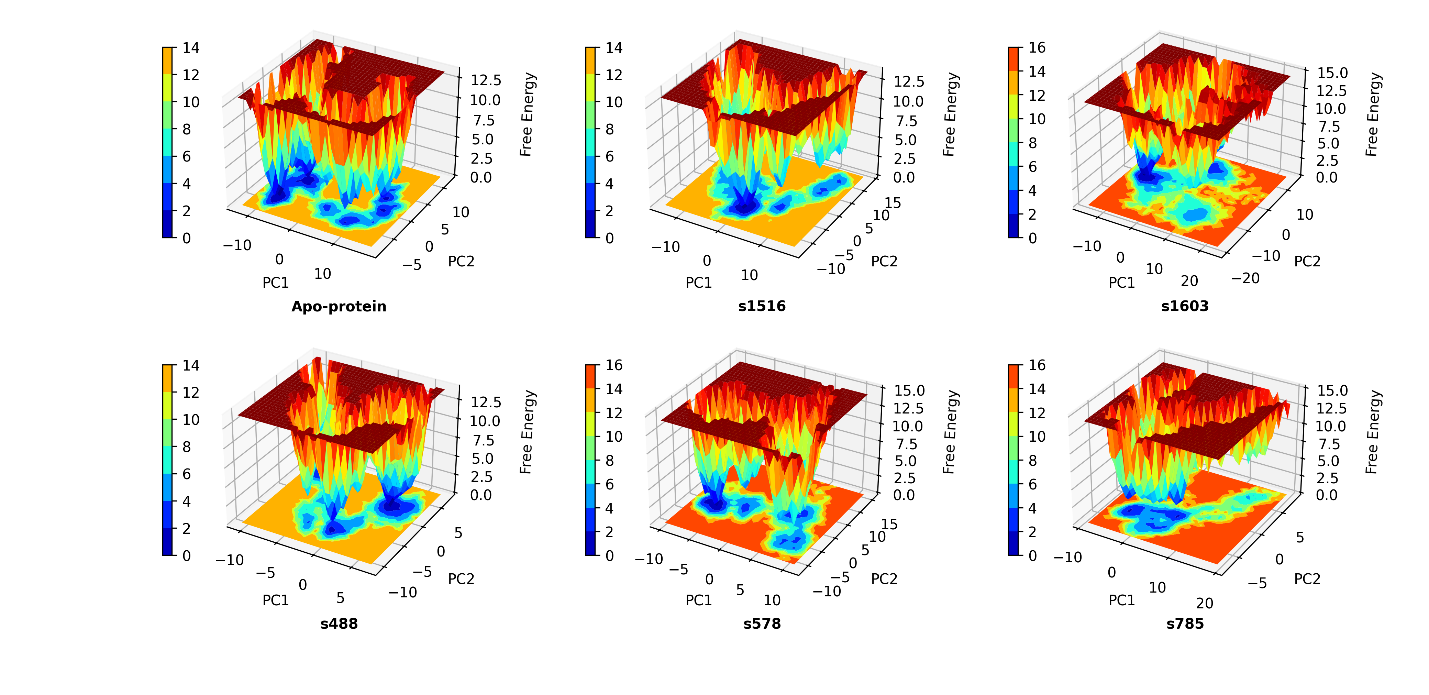


Fig S6 Free Energy Landscape (FEL) of Complexes formed by the selected peptides and Apo protein

**S7** **Post dynamic result of substituted peptides**

Fig S7 Post molecular dynamic simulation A) protein RMSD of substituted peptides B) RoG plot of substituted peptides C) RMSF of substituted peptides D) SASA of substituted peptides

**S8** **PCA of Complexes formed by the substituted peptides**

Fig S8 A) PCA plot of P1516 B) PCA plot of P785 C) PCA plot of P578 and D) PCA plot of 488

**S9** **Free Energy Landscape (FEL) of Complexes formed by the substituted peptides**


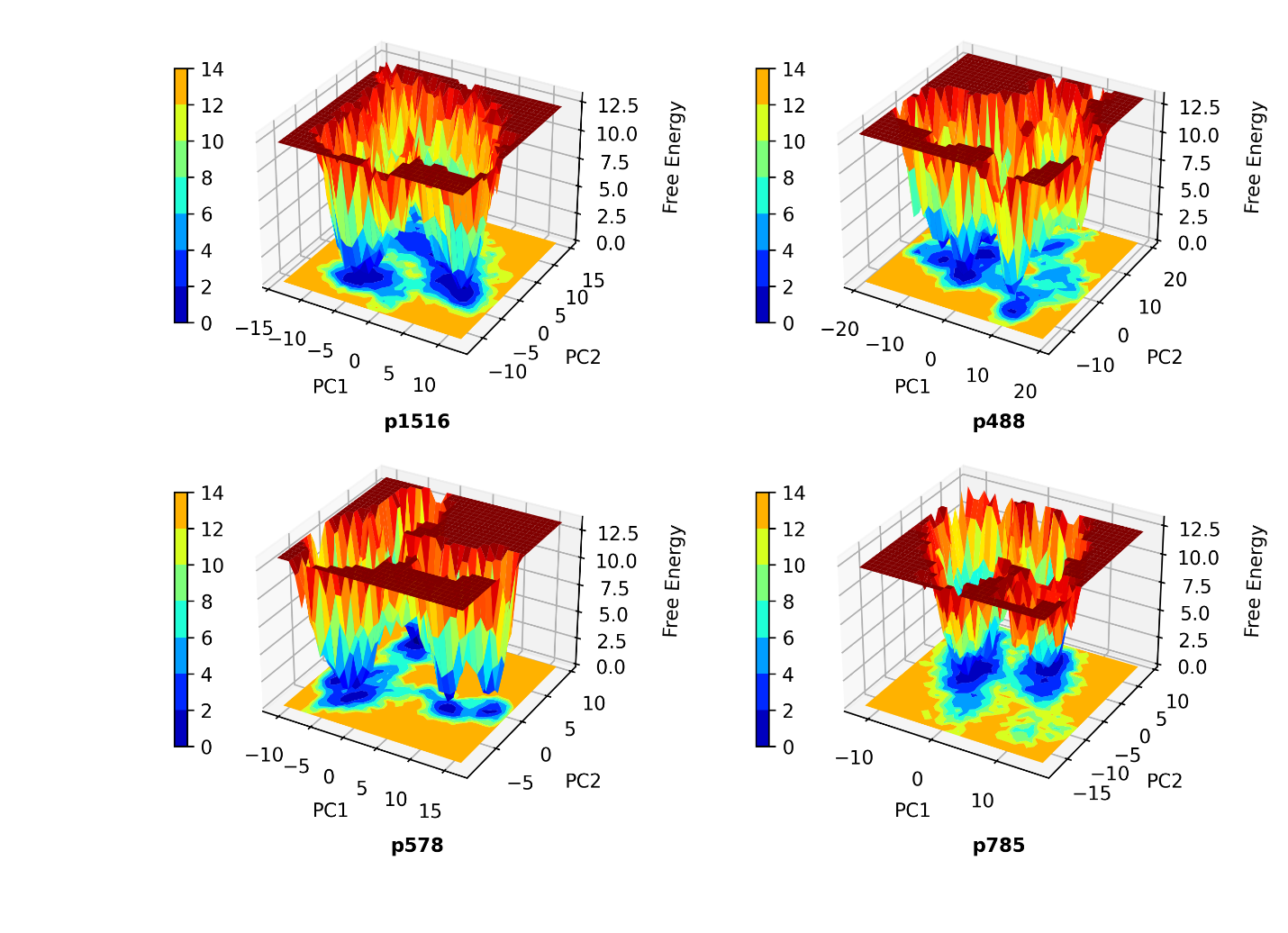


Fig S9 FEL of substituted peptides
